# Liver transplant score for prediction of biliary atresia patients’ survival following Kasai procedure

**DOI:** 10.1186/s13104-018-3498-z

**Published:** 2018-06-13

**Authors:** Gunadi, Timotheus Allen Gunawan, Galih Widiyanto, Amalia Yuanita, Nenny Sri Mulyani, Akhmad Makhmudi

**Affiliations:** 1 Pediatric Surgery Division, Department of Surgery, Faculty of Medicine, Public Health and Nursing, Universitas Gadjah Mada/Dr. Sardjito Hospital, Jl. Kesehatan No. 1, Yogyakarta, 55281 Indonesia; 2 Department of Child Health, Faculty of Medicine, Public Health and Nursing, Universitas Gadjah Mada/Dr. Sardjito Hospital, Yogyakarta, 55281 Indonesia

**Keywords:** Biliary atresia, Kasai procedure, Liver transplant score, Survival

## Abstract

**Objectives:**

Recently, a scoring system has been developed to predict which patients with biliary atresia (BA) who underwent a Kasai procedure should be considered for liver transplant. Here, we applied the scoring system to predict the survival of BA patients following the Kasai procedure at Dr. Sardjito Hospital, Yogyakarta, Indonesia from January 2012 to January 2016.

**Results:**

There were 26 patients, of whom 14 were males and 12 females. Outcomes of BA patients after the Kasai surgery were 15 survived and 11 died. There were significant associations between ascites and sepsis with the liver transplant score of ≥ 8 (*p* value = 0.006 and 0.014, respectively), whereas post-operative bilirubin level, ALT level, prothrombin time, cirrhosis, esophageal varices, portal hypertension, and cholangitis did not significantly correlate to the score. The patients with a score ≥ 8 have a relatively greater risk by 3.5-fold to die compared with patients with a score < 8, but it did not reach a significant level (*p* value = 0.13). In conclusions, the incidence of ascites and sepsis might predict the poor prognosis of BA patients following the Kasai procedure. Moreover, patients with a score ≥ 8 are prone to die after the Kasai surgery if they do not undergo a liver transplant.

## Introduction

Biliary atresia (BA), a destructive, inflammatory cholangiopathy affecting both the intra- and extrahepatic bile ducts, is the cause of death in children within 2 years of life due to biliary cirrhosis and liver failure if the Kasai procedure is not performed early in life [1, 2]. The incidence of BA varies among different populations, ranging from 1 in 5–10,000 live births in Taiwan [3] and Japan [4] to about 1 in 15–20,000 in mainland Europe [5], England, Wales [6] and North America [7].

The current treatment for BA is the Kasai operation in over 95% of infants diagnosed with BA, and the primary liver transplantation was used in only 2% of BA patients, particularly in England and Wales [8]. There are many predictor factors for survival of BA patients with native liver (i.e., without transplantation) after the Kasai procedure, however, recent studies still show conflicting results [9–11]. Furthermore, currently, a scoring system has been developed to predict which patients with biliary atresia (BA) who underwent a Kasai procedure should be considered for liver transplantation [12]. Therefore, we applied the scoring system to predict the survival of BA patients with native liver (i.e., without transplantation) following the Kasai procedure.

## Main text

### Methods

#### Patient samples

We conducted a retrospective study of infants with BA at the Pediatric Surgery Division, Department of Surgery, Dr. Sardjito Hospital in Yogyakarta, Indonesia from January 2012 to January 2016. Dr. Sardjito Hospital is a University Teaching Hospital and as a tertiary referral center, it serves urban and rural populations from Yogyakarta province [13–15].

From January 2012 to January 2016, we evaluated 31 BA cases who underwent the Kasai procedure, of whom five participants were excluded because of incomplete laboratory data. Twenty-six patients had adequate data for analysis, consisting of 14 males and 12 females, corresponding to a sex ratio of 1.2:1 (Table 1).

**Table 1:** Characteristics of biliary atresia patients following Kasai procedure and its association with liver transplant scoring system

Characteristics	n (%); mean ± SD (normal range)
Gender	
Male	14 (54)
Female	12 (46)
Age at diagnosis (days)	126 ± 77.9
Biliary atresia type	
Type 1	0
Type 2a	6 (23)
Type 2b	2 (8)
Type 3	18 (69)
Pre-operative body weight (kg)	5.5 ± 2.4
Pre-operative laboratory findings	
Total bilirubin (mg/dL)	10.8 ± 5.4 (≤ 1.0)
Direct bilirubin (mg/dL)	8.4 ± 2.7 (0–0.2)
Alanine aminotransferase (ALT) (U/L)	140 ± 75.1 (≤ 41)
Aspartate aminotransferase (AST) (U/L)	231.7 ± 98.0 (≤ 40)
Alkaline phosphatase (ALP) (U/L)	568.5 ± 249.8 (≤ 462)
Gamma glutamyl transferase (GGT) (U/L)	544.4 ± 341.9 (7–64)
Albumin (g/dL)	3.3 ± 0.6 (3.9–4.9)
International normalized ratio (INR)	1.9 ± 1.6 (0.9–1.1)
White blood cells (×10^3^/μL)	13.9 ± 8.3 (4.5–13.5)
Neutrophil (%)	41.0 ± 14.1 (35.0–65.0)
Lymphocyte (%)	42.5 ± 19.4 (23.0–53.0)
Pre-operative portal hypertension features	
Esophageal varices	3 (12)
Splenomegaly	10 (38)
Platelet (×10^3^/μL)	315.4 ± 176.8 (150–450)
Ascites	15 (58)
Age at Kasai procedure performed (days)	
< 60	2 (8)
≥ 60	24 (92)
Histopathological findings of liver biopsy	
Liver cirrhosis	11 (42)
Non-liver cirrhosis	15 (58)
Sonography findings	
Undetectable gallbladder	11 (42)
Triangular cord sign	5 (19)
Outcomes	
Survived	15 (58)
Died	11 (42)
Cause of death	
Septic shock	2 (18)
Pulmonary edema	2 (18)
Hemorrhagic shock	1 (9)
Aspiration pneumonia	2 (18)
Hepatopulmonary syndrome	1 (9)
Multiple organ dysfunction syndrome	3 (27)

Characteristics	Liver transplant score		p value	OR (95% CI)
	< 8	≥ 8		
Gender				
Male	6	8	0.23	2.7 (0.5–13.2)
Female	8	4		
Age at Kasai procedure performed (days)				
≥ 60	12	12	0.31	5.0 (0.2–115.1)
< 60	2	0		

The Ethical Committee of Faculty of Medicine, Universitas Gadjah Mada/Dr. Sardjito Hospital gave approval for this study (KE/FK/528/EC/2015).

#### Liver transplant scoring system

The liver transplant scoring consists of nine factors that include: post-operative serum bilirubin (0 ≤ 2.0 mg/dL, 1 = 2.0–4.0 mg/dL, 2 ≥ 4.0 mg/dL); post-operative alanine aminotransferase (ALT) (0 ≤ 40 U/L, 1 = 40–80 U/L, 2 ≥ 80 U/L); prothrombin time (0 ≤ 4 s prolonged, 1 = 4–6 s, 2 ≥ 6 s); cirrhosis, ascites, esophageal varices, and portal hypertension (0 = absent, 1 = present); and cholangitis and sepsis (0 = none, 1 = once, 2 = recurrent) [12]. We took the above data at 1 month following Kasai procedure. The BA patients with a score of ≥ 8 indicate the need for liver transplant. We classified our patients into a score of < 8 group (i.e., good prognosis) and a score of ≥ 8 group (i.e., poor prognosis).

#### Statistical analysis

Data are presented as number and percentages for categorical variables. Chi square test and logistic regression were used to evaluate the association of prognostic factors and outcome of BA patients after the Kasai procedure. IBM SPSS Statistics version 16 (SPSS Chicago, IL, USA) was used for statistical analysis.

### Results

**Fig. 1:**
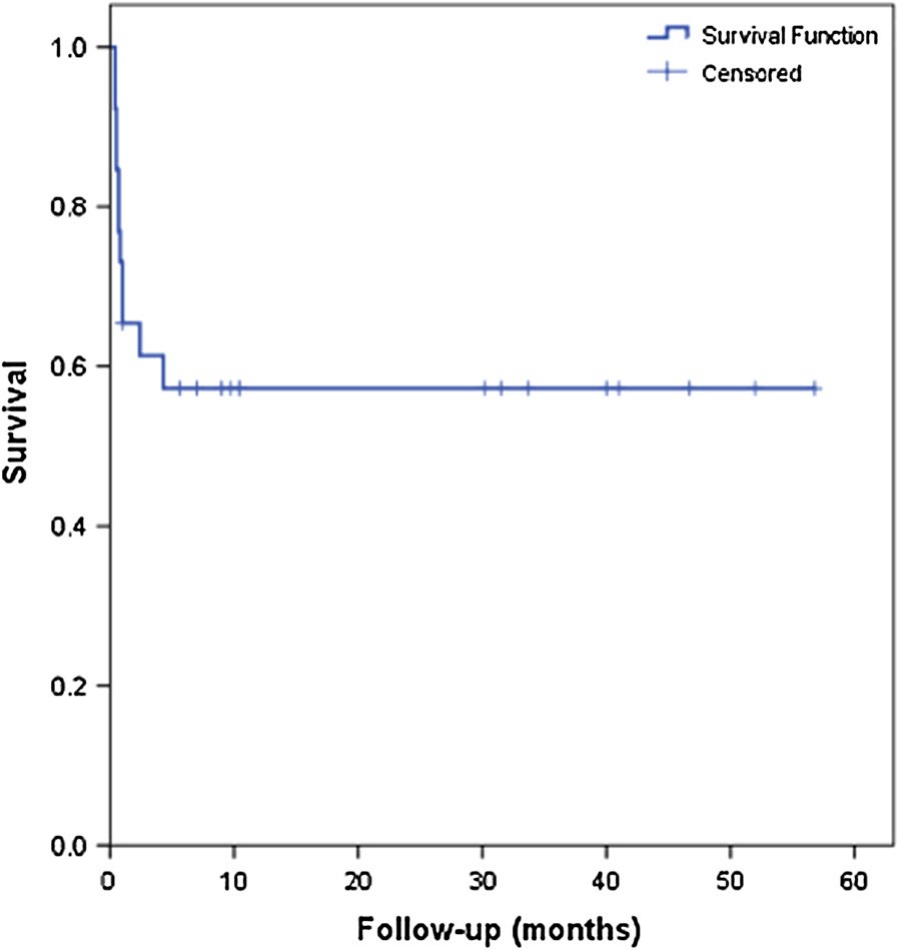
Survival curve of biliary atresia patients following Kasai procedure

We analyzed 26 BA patients who underwent the Kasai procedure during a 4-year study period, with mean age at diagnosis of 126 ± 77.9 days. Pre-operative sonography showed a triangular cord sign and undetectable gallbladder in 5 and 11 infants with BA, respectively (Table 1). All pre-operative clinical and laboratory finding were shown in the Table 1. The outcomes of BA patients after the Kasai surgery were 15 survived and 11 died. The mean follow-up time after Kasai procedure was 23.5 ± 18.4 months. Furthermore, the survival curve of biliary atresia patients following Kasai procedure at our institution was shown in the Fig. 1.

During the study period, the number of newborns in Yogyakarta Province was 185,144 [16]. Therefore, the estimated incidence of BA in Yogyakarta, Indonesia during the study period was ~ 1:7000.

Our first analyses involved the association of BA patient’s characteristics with the scoring system. Male patients and age of ≥ 60 days for the Kasai procedure showed an increased risk of 2.7- and five-fold to have a score of ≥ 8 (i.e., poor prognosis), but it did not reach a significant level (*p* value = 0.23 and 0.31, respectively) (Table 1).

Next, we analyzed the impact of prognostic factors on the liver transplant scoring system. Among the nine factors, only ascites and sepsis revealed a strong association with the score of ≥ 8 (*p* = 0.006 and 0.014, respectively) with OR of 27.5 (95% CI 2.6–289.1) and 9.0 (95% CI 1.4–58.4), respectively, whereas the post-operative bilirubin level, ALT level, prothrombin time, cirrhosis, esophageal varices, portal hypertension, and cholangitis did not show statistically significant correlations (Table 2).

**Table 2:** Liver transplant scoring system and its impact on survival of biliary atresia patients after Kasai surgery

Liver transplant scoring factor	Liver transplant score		*p* value	OR (95% CI)
	< 8	≥ 8		
Post-operative bilirubin level (mg/dL)				
> 4	10	10	0.48	2.0 (0.3–13.5)
≤ 4	4	2		
Post-operative ALT level (U/L)				
> 80	4	7	0.13	13.5 (0.7–17.9)
≤ 80	10	5		
Prothrombin time (s prolonged)				
> 6	14	12	0.94	0.86 (0.02–46.7)
≤ 6	0	0		
Cirrhosis				
Present	5	6	0.46	1.8 (0.4–8.7)
None	9	6		
Ascites				
Present	4	11	0.006	27.5 (2.6–289.1)
None	10	1		
Sepsis				
Present	5	10	0.014	9.0 (1.4–58.4)
None	9	2		
Cholangitis				
Recurrent	1	0	0.54	0.4 (0.01–9.7)
Once/none	13	12		
Portal hypertension				
Present	1	4	0.12	6.5 (0.6–68.9)
None	13	8		
Esophageal varices				
Present	0	1	0.43	3.8 (0.1–101.8)
None	14	11		

Liver transplant score	Outcomes		*p* value	OR (95% CI)
	Survived	Dead		
≥ 8	5	7	0.13	3.5 (0.7–17.9)
< 8	10	4		

To determine the effect of a score ≥ 8 on the survival of BA patients following the Kasai procedure, we analyzed the observed number of survived and dead patients with respect to the scoring system. The results shown in Table 2 clearly demonstrate that the patients with a score ≥ 8 have a relatively greater risk by 3.5-fold to die compared with the patients with a score < 8, but statistically, the results did not reach a significant level (*p* = 0.13) (Table 2).

There were several causes of death in our cohort patients, such as septic shock, pulmonary edema, hemorrhagic shock, aspiration pneumonia, hepatopulmonary syndrome, and multiple organ dysfunction (Table 1).

### Discussion

We present new data on Indonesian BA patients, largely with type 3, that demonstrate a similar incidence with other Asian populations [3, 4], but are higher than Caucasian studies [5–8]. Most patients were referred to our hospital beyond 4 months of age (Table 1). This late age is compatible with previous reports that indicate that most BA cases in developing countries present late: 5% cases were seen < 60 days of age, 40% between 2 and 3 months, 30% between 3 and 4 months, and 25% presented > 4 months of age [17].

Overall survival of our series was nearly 60%, which is comparable with other reports [17]. This survival rate might relate to the fact that our BA patients underwent the Kasai procedure by experienced pediatric surgeons and at a qualified care center. Our hospital is a referral hospital for biliary atresia management and has performed successful Kasai procedures for almost 15 years. It has been proposed that one of the most important prognostic factors for the Kasai procedure outcome are the surgeons’ experience and the quality of the care center at which the Kasai surgery is performed [18].

Other important prognostic factors for the Kasai procedure outcome is younger age at the time the Kasai surgery is performed [19]. In our series, although not statistically significant, the BA patients with the age of ≥ 60 days for the Kasai procedure showed a higher risk (fivefold) for poor prognosis compared with those of the age of < 60 days. Many studies showed that the patients’ outcomes were better if the surgery was performed at an earlier age of life, however, it is still debatable when is the appropriate time to perform the Kasai procedure to get a good outcome [11, 19–21].

One previous study revealed that a score of ≥ 8 had a high sensitivity (96.9%) and specificity (89.5%) for predicting the need for liver transplantation [12]. They also included the complications of the Kasai procedure including cholangitis, liver cirrhosis, and sepsis, into the scoring system since a good initial surgical outcome does not rule out the subsequent episode of progressive jaundice, recurrent cholangitis, portal hypertension, growth and nutritional failure, and end-stage liver disease [12]. Our results showed that the ascites and sepsis are strong prognostic factors for poor prognosis after the Kasai surgery. Ascites is one of the signs for liver cirrhosis, while sepsis is one of the Kasai surgery complications. In addition, according to the Netherland database, related research revealed that sepsis is a major contributor for pre-transplant mortality of BA patients. They strongly suggested that the improvement of the prognosis of BA patients might be achieved by undergoing liver transplant [22]. Furthermore, we have now been preparing to develop a liver transplant center in our institution, including the living donor program.

Although not statistically significant, our series showed that the patients with a score of ≥ 8 have a relatively higher possibility to have a poor prognosis (i.e., dead). It supports the proposed hypothesis that the patients with a score of ≥ 8 should undergo a liver transplant [22]. Furthermore, one previous cohort study demonstrated that the survival of patients with bilirubin ≥ 2 mg/dL 3 months after Kasai surgery without liver transplant was only 20%. The findings showed that they had a higher risk to suffer from poor weight gain, hypoalbuminemia, and coagulopathy [10].

### Conclusions

The incidence of ascites and sepsis might predict the poor prognosis of BA patients following the Kasai procedure. Moreover, patients with a score ≥ 8 are prone to die after Kasai surgery if they do not undergo a liver transplant.

## Limitations

We only extracted data from the medical records (retrospective study). Furthermore, it should be noted that the small sample size, becoming a weakness of our study, implies that a much larger cohort of patients needs to be ascertained to clarify our findings. In addition, this is a mono-institutional study, therefore caution should be also taken when generalizing about the findings.

## Abbreviation

ALT: alanine aminotransferase
ALP: alkaline phosphatase
AST: Aspartate aminotransferase
BA: biliary atresia
GGT: gamma glutamyl transferase
INR: international normalized ratio
